# Human errors and factors that influence patient safety in the pre-hospital emergency care setting: Perspectives of South African emergency care practitioners

**DOI:** 10.4102/hsag.v27i0.1798

**Published:** 2022-04-29

**Authors:** Mugsien Rowland, Anthonio Adefuye

**Affiliations:** 1 Department of Emergency Medical Care, Faculty of Health Sciences, Nelson Mandela University, Port Elizabeth, South Africa; 2 Department of Health Sciences Education, Faculty of Health Sciences, University of the Free State, Bloemfontein, South Africa

**Keywords:** emergency care practitioners, human error, patient safety, pre-hospital emergency care setting, South Africa

## Abstract

**Background:**

Delivering pre-hospital emergency care has the potential to be hazardous. Despite this, little is known about the factors that precipitate human errors and influence patient safety in the pre-hospital care setting, in contrast to in-hospital care. Similarly, limited report on patient safety and human error issues in the pre-hospital emergency care setting exist in South Africa.

**Aim:**

This study investigated the perspectives of emergency care personnel (ECP) in South Africa on the types of human errors and factors that precipitate human errors that influence patient safety in the pre-hospital emergency care setting in South Africa.

**Setting:**

This study was conducted in the pre-hospital emergency care environment in South Africa.

**Methods:**

This research was designed as an exploratory study that made use of a semi-structured questionnaire administered to 2,000 emergency care personnel.

**Results:**

A response rate of 76% was obtained. According to the participants, errors relating to poor judgement, poor skill or knowledge, fatigue, and communication, and individual error are common during pre-hospital care. Inadequate equipment, environmental factors, and personal safety concerns were reported as some of the factors that influence patient safety in the pre-hospital emergency care setting.

**Conclusion:**

Implementation of strategies that enhances education and training, clinical skill development, teamwork skills, fatigue management, and leadership skills can help prevent some of the errors identified in this study.

**Contribution:**

This study identifies the types of human errors, and factors that precipitate human errors that influence patient safety in the pre-hospital emergency care setting in South Africa.

## Introduction

The healthcare professional, being human, has been described as a small, but probably the most error-prone part of a modern healthcare system that is dependent on an array of non-human systems (Bari, Khan & Rathmore 2016; Bashir et al. 2019). There is a growing realisation that human error adversely influences patient safety and contributes significantly to morbidity and mortality in a modern healthcare system (Bashir et al. 2019). Preserving patient safety in contemporary medical practice is a global problem; it has been estimated that 10% of patients admitted to a hospital are exposed to harmful incidents, and 2% die because of medical error (attributed to human error) (Runciman & Walton 2007). While these estimates might be true for developed countries, there is currently very limited literature on the impact of error on patient safety in developing countries (Bashir et al. 2019).

In a retrospective review of patients’ hospital records across eight developing countries (including South Africa), Wilson et al. (2012) estimated that the medical error rate in developing countries was 8.2% of hospital admissions. Recent reports from public hospitals across Gauteng, the most populated province in South Africa, shows a steady increase in medical errors leading to unintended harm to patients between 2019 and 2020 (Molelekwa 2021). Most reports and investigations into human error and patient safety in the healthcare setting are hospital-based; however limited similar studies are conducted in the pre-hospital emergency care setting (Hagiwara et al. 2019).

Pre-hospital emergency medical care can be provided anywhere, including at the roadside, in a home, or in a public setting, with associated threats to safety that are unspecified, unpredictable, and/or uncontrollable (Patterson & Yealy 2019). In this milieu, the emergency care personnel (ECP), health care professionals skilled in providing pre-hospital emergency care, must think, move, and react swiftly to reduce threats to life and limb, and to stabilise the patient quickly (Patterson & Yealy 2019).

Furthermore, delays, interruptions, or distractions are common in this high-risk environment, and may contribute to errors in judgment, medication choices and delivery, or in executing procedures (Patterson & Yealy 2019). Emergency care personnel in South Africa are not exempted from these errors. Despite this potential, yet known, threats, limited reports on patient safety and human error issues in the pre-hospital care setting exist in South Africa.

In a Canadian study, errors due to clinical reasoning and decision-making were regarded as the most important factors that influence patient safety in a pre-hospital emergency care setting (Bigham, Brooks & Maher 2011b). In addition, Makkink, Stein and Bruijns (2021) reports that communication and process barriers are important factors that affect patient safety during pre-hospital emergency care. This study investigated the perspectives of ECPs in South Africa on the types of human errors and factors that precipitate human errors that influence patient safety in the pre-hospital emergency care setting in South Africa. Identifying these errors and factors that precipitate them, will assist emergency care providers in developing strategies to mitigate such errors.

## Methods

This research was designed as an exploratory study that utilised a survey tool to obtain both quantitative and qualitative data.

### Study setting

The Emergency Medical Service (EMS) profession in South Africa owes its existence to the short courses (vocational qualification courses) such as the 2-weeks Basic Ambulance Assistant (BAA), 12-week, intermediate life support (ILS), Ambulance Emergency Assistant (AEA), and a 4-month Advanced Life Support (ALS), Critical Care Assistant (CCA) (National Department of Health: Republic of South Africa 2017). However, over the past two decades, the profession has undergone continuous reviews and reforms, paving the way for the current structured three-tiered Emergency Care Qualification Framework (ECQF), adopted by the National Department of Health (National Department of Health: Republic of South Africa 2017; Vincent-Lambert 2011, 2015). Depending on their qualifications, skills and competencies, the Health Professions Council of South Africa (HPCSA) categorise and register ECPs as Basic Life Support (BLS) provider, ILS provider or ALS provider, respectively.

### Study population

The study population comprised ECPs in South Africa (employed in either the state or private sector) who were, at the time of the study, registered with the HPCSA. The full list and the contact details (e-mail addresses) of registered ECPs were obtained, with permission, from the HPCSA. At the time of this study, the total number of registered ECPs with registered email addresses, obtained from HPCSA, was *N* = 24 970 (sample frame). The estimated minimum sample size was calculated as *n* = 1000 (at 99% confidence level [CL] and margin of error [MOE] 0.04). Simple random sampling technique was utilised and the target population comprised *n* = 2000 ECPs. The study sample consists of respondents who consented to complete the questionnaire and thereby participate in the study.

### Questionnaire survey

The semi-structured questionnaire was developed by the researchers using questionnaire items identified from prior studies, (Bigham et al. 2011a; Hagiwara et al. 2019) following a thorough literature review and content analysis. The literature review included medical subject headings search terms, such as medical error, human error in pre-hospital care, medical error in pre-hospital care, patient safety, and patient safety in pre-hospital care.

The databases used to access articles were Google Scholar, MEDLINE, PubMed, CINAHL, SABINET, Science Direct, and Directory of Open Access Journals. Concepts were identified to formulate both open-ended and closed-ended questions; the closed-ended questions were answered using adapted Likert-scale ranking. The open-ended questions allowed for the investigation of perceptions and provided contextual depth to the data collected. The questionnaire consisted of five sections.

The first section, Section A, focused on participants’ demographic information (i.e. data on participants’ age, gender, registration cadre, employment profile and province of employment) and generated quantitative data.

Section B, an open-ended section, obtained information about participants’ perspectives on the different types of human error in the pre-hospital care setting and generated qualitative data.

Section C focused on identifying factors participants perceived as contributing to human error in the pre-hospital care setting and generated qualitative data.

In Section D, the statement, ‘List some of the factors influencing patient safety in the pre-hospital care setting’, was used to obtain qualitative data on participants’ views on factors that may influence patient safety in the pre-hospital care setting.

Section E obtained information (quantitative data) on aspects of organisational culture and attitudes towards patient safety and human error in the pre-hospital care setting using a five-point Likert scale with the options ‘Never’, ‘Once in a while’, ‘About half the time’, ‘Most of the time’ and ‘Always’. For the purpose of analysis, these options were reduced to three-point Likert scale, as follows: ‘Never’ + ‘Once in a while’ = ‘Never’; ‘Most of the time’ + ‘Always’ = Always; and ‘About half the time’.

The questionnaire was administered electronically via the QuestionPro survey management system. A cover letter that elucidated the objectives of the study, as well as the conditions for study participation, was sent by e-mail to all potential participants. By clicking on the link to the questionnaire, participants automatically agreed tacitly to participate in the study.

A waiting period of 1 week from the date of delivery was given for participants to complete the survey, after which repeated electronic reminders to complete the questionnaire – one per week – were sent for three consecutive weeks. The survey was conducted from 26 January 2021 to 26 February 2021.

### Pilot study

A pilot study was executed prior to the official start of data collection, to test the suitability of the study’s design and methods, the chosen data collection method, and the overall structure of the questionnaire. The pilot study involved 10 ECPs. An e-mail that contained an information sheet and a request to participate in the pilot study, as well as an electronic links to the questionnaire, were sent to each participant. The findings of the pilot study confirmed the adequacy of the chosen data collection method and the overall structure of the questionnaire. The participants in the pilot study did not recommend changes to the structured questionnaire. They estimated the total time needed to complete the questionnaire to be approximately 30 min. The data from the pilot study was not included in the main study.

### Validity

Questionnaire validity was achieved by comparing the questionnaire elements with those of similar studies and by conducting a pilot study. In addition, a faculty evaluation committee subjected the questionnaire for review and approval (Ringsted, Hodges & Scherpbier 2011).

### Reliability

The closed-ended questions in the questionnaire were grouped into subsets and analysed for reliability by using Cronbach’s alpha following data collection (Bland & Altman 1997; Devellis 2016; Taber 2018). Cronbach’s analysis for the subset of questions in the questionnaire ranged from 0.57 to 0.78, thus, suggesting that the items within the subsets were related to one another and would provide reliable answers to the questions they were designed to investigate.

### Data analysis

Statistical analysis of all numerical data was performed by using the statistical software package TIBCO Statistica^TM^ version 13.5.0.17. Results are presented in contingency tables as frequencies and percentages. Chi-squared test and Cramér’s V were used to examine the existence and the strength of an association between cross-tabulated variables. Responses to open-ended questions were analysed qualitatively using Atlas.ti 8.3 software (Scientific Software Development, Germany). Iterative inductive coding was done with the assistance of a co-coder, and attention was paid to the emerging patterns and themes. Briefly, participants’ responses were read and re-read by the researchers to familiarise themselves with the content; unit of meaning (codes) were then generated from the data, the generated units of codes were grouped into categories, and major categories grouped to form themes (Kekeya 2016; Thomas 2006). Spearman’s rho correlation was used to analyse the strength of association between factors that influence human error, and the perceived types of human error. Identified themes were first converted to numerical variables using code or theme frequency count. Spearman’s rho correlation was then used to measure the degree of association between the two variables.

### Ethical considerations

The Health Sciences Research Ethics Committee of the Faculty of Health Sciences at the University of the Free State provided ethical clearance for this study, ethical clearance number: HSD2020/0462/2807.

## Results

### Participant demographics

Out of 2000 questionnaires distributed, 1510 were returned, giving a response rate of 76%.

### Gender

Of the participants, 62.6% (*n* = 909) were men and 37.4% (*n* = 542) were women (ratio 2:1).

### Age and years of experience

The median age was 37.4 years (± SD = 1.14 years), with the majority (40.3%; n = 592) within the age bracket 25–34 years and having worked for an average of 11.2 years (± SD = 8.4).

### Registration

Participants’ registration cadres are as follows: BAA (38.6%; *n* = 566); AEA (29.4%; *n* = 432); Emergency Care Practitioner (ECP) (9.4%; *n* = 138); Emergency Care Technician (ECT) (9.1%; *n* = 134); CCA (8.4%; *n* =124); Emergency Care Assistant (ECA) (0.2%; *n* = 3); and Other (4.8%; *n* = 71).

### Nature of employment

Regarding the nature of their employment, the majority (83.4%; *n* = 1186) of the participants reported that they worked as operational emergency care practitioners, 11.3% (*n* = 160) worked as emergency care support staff, and only 5.3% (*n* = 5) worked in EMS communications.

### Sector and location of workplace

The majority (53.1%; *n* = 736) of the participants were employed in the private sector, while 46.9% (*n* = 649) worked in the public sector. Eight-hundred-and-ninety-six (62.9%) participants indicated that their EMS base is located in an urban area, 20.1% (*n* = 286) indicated that they work in rural areas, and 17.1% (*n* = 243) worked in semi-rural areas.

#### Participants’ perspectives on types of human error in the pre-hospital emergency care setting

Analysis of the participants’ responses to the types of human error that occur in the pre-hospital environment yielded five dominant themes, namely poor judgement error, poor skill or knowledge-based error, fatigue-related error, individual error, and communication error.

### Poor judgement error

The majority of the participants reported that poor judgement errors often occur as misdiagnoses, and incorrect medication or dosages, as reported by quotes #1 to #3:

- #1 ‘Incorrect interpretation of diagnosis’
- #2 ‘Wrong drug dosages’
- #3 ‘Misdiagnosis, wrong dose/drug’

### Poor skill or knowledge-based error

According to the participants, poor skill or knowledge-based error in the pre-hospital care setting can represent as poor clinical reasoning and poor patient management (cf. quotes #4–#6):

- #4 ‘Lack in clinical reasoning leading to wasted time on scene’
- #5 ‘Management related errors’
- #6 ‘incorrectly managing a patient due to lack of knowledge’

In addition, participants listed lack of proper training and failure to attend continuous professional development programmes as among the factors that can precipitate poor skill-based errors (cf. quotes #7 to #10):

- #7 ‘Not doing refresher courses’
- #8 ‘Lack of proper training’
- #9 ‘lack of adequate training’
- #10 ‘Not doing clinical updates’

### Fatigue-related error

Participants reported that fatigue-related errors often present as lapses in concentration, leading to mistakes when attending to patients (cf. Quotes #11 and #12); fatigue is attributed to work overload, lack of rest, stress and burnout, as reported by quotes #13 to #19:

- #11 ‘lapse in concentration i.e. mistakes’
- #12 ‘Loss of concentration’
- #13 ‘Poor concentration’
- #14 ‘overloading of work’
- #15 ‘overwork/tired’
- #17 ‘No resting enough before shift’
- #18 ‘stress when dealing with difficult situations, panic’
- #19 ‘Burnout’

### Individual error

Participants indicated that bad attitudes and behaviours, lack of confidence, and fear leading to negligence in practice constitute some of the human errors occurring in the pre-hospital emergency care setting (cf. Quotes #20 to #25):

- #20 ‘Lack of confidence’
- #21 ‘Fear’
- #22 ‘Bad attitudes’
- #23 ‘poor practitioner attitude’
- #24 ‘Negligence’
- #25 ‘Carelessness, Attitude’

### Communication error

As described by the participants, communication errors in the pre-hospital emergency care setting could occur because of poor or miscommunication between ECPs, language barriers between the practitioners and patients, and failure to communicate with patients (cf. Quotes #26 to –#33):

- #26 ‘Lack of effective communication’
- #27 ‘Miscommunication’
- #28 ‘Communication barriers’
- #29 ‘not communicating with the patient’

#### Factors participants perceive as contributing to human error in the pre-hospital emergency care setting

Analysis of participants’ responses regarding factors that are perceived to contribute to human error in the pre-hospital emergency care setting generated eight emerging themes, namely work-related fatigue and/or stress, insufficient education and training, insufficient clinical knowledge and experience, unsafe work environment, poor communication skills, being overconfident, poor leadership and management practices, and intimidation by and pressure from senior colleagues.

### Work related fatigue and/or stress

The majority of the participants reported that work-related fatigue and/or stress represent a major contributing factor to human error in the pre-hospital emergency care environment (cf. Quotes #30 to #34). In addition, work-related fatigue or stress was found to correlate positively with fatigue-related error (*r* = 0.24; *p* ≤ 0.01):

- #30 ‘Fatigue’
- #31 ‘Stress at work’
- #32 ‘Exhausted/burnt out personnel’
- #33 ‘Stress induced freezing’
- #34 ‘Fatigue and practitioner burnout’

### Insufficient education and training

Participants also indicated that insufficient training as well as not attending continuous development programmes could contribute to human error in the pre-hospital emergency care setting as shown in quotes #35 to #39. A high positive correlation was found between insufficient education and training and poor skill or knowledge-based error (*r* = 0.92; *p* ≤ 0.01):

- #35 ‘Poor training’
- #36 ‘Lack of knowledge and training’
- #37 ‘Lack of education/training/revision’
- #38 ‘Not enough training’
- #39 ‘Lack of continuous development’

### Insufficient clinical knowledge and experience

Limited clinical knowledge, as well as lack of experience in pre-hospital emergency care, were also suggested by the participants as factors that may lead to human error when attending to patients in the pre-hospital setting (cf. Quotes #40 to #47). This factor was found to correlate positively and significantly with poor skill or knowledge-based error (*r* = 0.83; *p* ≤ 0.01):

- #40 ‘Lack of exposure or lack of experience’
- #41 ‘Not knowing the work’
- #42 ‘Lack of knowledge’
- #43 ‘Lack of clinical knowledge’
- #45 ‘Insufficient skills set or knowledge’
- #46 ‘Lack of adequate experience’
- #47 ‘No knowledge’

### Unsafe work environment

Participants of this study, furthermore, indicated that fear for personal safety and stress associated with working in an unsafe environment contribute to human error in the pre-hospital setting, as exemplified by quotes #48 to #54:

- #48 ‘Unsafe environment’
- #49 ‘Danger… crime… gangs… rioting’
- #50 ‘Extreme stress due to threat to your life’
- #51 ‘Fear of being mugged end up doing mistakes if working in the red zone’
- #52 ‘Red zones. Safety’
- #53 ‘Environmental danger’
- #54 ‘Fear of personal safety’

### Poor communication skills

Miscommunication, inability to communicate effectively, and lack of communication was cited by the participants as factors that can contribute to human error in the pre-hospital emergency care setting (cf. Quotes #55 to #59). Similarly, the factor, poor communication skills correlates positively with communication error (*r* = 0.84; *p* ≤ 0.01):

- #55 ‘Poor communication’
- #56 ‘Inability to communicate effectively’
- #57 ‘Lack of communication’
- #58 ‘Communication breakdown’
- #59 ‘Poor communication skills’

### Overconfidence

A ‘know-it-all’ attitude or feeling of being overconfident about one’s clinical skills was also identified as one of the factors that may contribute to errors in pre-hospital care, as shown by quotes #60 to #62:

- #60 ‘Over confidence’
- #61 ‘Knowing it all’
- #62 ‘Be over confident’

### Poor leadership and management practices

Participants, furthermore, reported that a lack of good leadership and management practices at an emergency scene could contribute to errors during pre-hospital care, as highlighted by quotes #63 to #66:

- #63 ‘Poor Leadership Practices’
- #64 ‘Poor Management Practice’
- #65 ‘Without clear leadership or direction’
- #66 ‘Collapse of the chain of command…’

### Intimidation by and pressure from senior colleagues

Lastly, participants were of the opinion that intimidation by and pressure from senior colleagues can also precipitate human error during pre-hospital emergency care (cf. Quotes #67 to #69):

- #67 ‘Pressure from Superiors to meet timelines’
- #68 ‘Fear of higher qualified practitioners’
- #69 ‘Intimidation by senior medics’

## Factors perceived by participants as influencing patient safety in the pre-hospital emergency care setting

**FIGURE 1:**
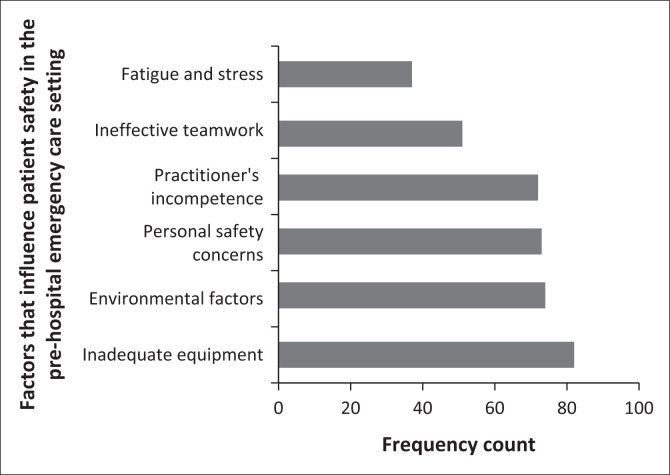
Participants’ views on factors that influence patient safety in pre-hospital emergency care setting.

In this section of the questionnaire, participants were asked to list factors that, in their own opinion, influence patient safety in the pre-hospital emergency care setting. Analysis of participants’ responses generated six dominant themes. Identified themes together with their frequency count are presented in Figure 1. Inadequate equipment, environmental factors, personal safety concerns and practitioners’ incompetence are the top four influencers of patient safety in the pre-hospital emergency care setting.

## Organisational culture and attitudes about patient safety and human error in the pre-hospital care setting

### Protocol for managing medical errors

First, participants were asked to indicate whether their organisation has a protocol for managing medical errors. Results as shown in Table 1 reveal a weak but significant association between the type of workplace and the presence of a protocol for managing medical error. The majority (65.7%) of the respondents who work in the public sector indicated that they do not have a protocol for managing medical error at their workplace (*χ*^2^ = 28.8; Cramer’s V = 0.2; *p* < 0.001).

**TABLE 1:** Availability of protocol for managing medical error at workplace (*n* = 742).

Type of workplace	Protocol for managing medical error at the workplace	
	Yes (%)	No (%)
Public sector	42.2	65.7
Private sector	57.8	34.3

### Training to ensure patient safety during pre-hospital emergency care

In this regard, participants were asked to indicate how often they received training to ensure patient safety during pre-hospital emergency care. The results show that the majority of the participants working in either the private (53.7%) or public (65.1%) sector has never received training on patient safety (*χ*^2^ = 14.1; Cramer’s V = 0.14; *p* < 0.007) (Table 2).

**TABLE 2:** How often participants received training on patient safety (*n* = 754).

Frequency of training on patient safety	Type of workplace	
	Public (%)	Private (%)
Never	65.1	53.7
About half the time	11.9	14.0
Always	23.0	32.3

## Discussion

Findings of this study about the male to female ratio – 2:1 – are consistent with the findings by other studies and confirms that the pre-hospital emergency medical care environment in South Africa is a male-dominated field (Mothibi, Jama & Adefuye 2019). This finding suggests that there may be a gender bias in the EMS profession.

The median age of 37.4 years is consistent with the findings by Mothibi et al. (2019), and suggest that a relatively young population dominates the EMS profession in South Africa (Mothibi et al. 2019). Historically, the EMS profession in South Africa owes its existence to short vocational qualification courses, such as the BAA, AEA, and CCA. An increase in the number of training colleges and institutions accredited by the HPCSA to offer short courses in the early nineties increased the numbers of ECPs trained.

Thus, by 2018, the majority of pre-hospital ECPs registered with the HPCSA were BAA and AEA practitioners (Sobuwa & Christopher 2019). This change is reflected in the findings of this study, which report that the majority (68%) of participants were registered as BAA and AEA. In South Africa, more healthcare workers are employed in the private sector than in the public sector (Econex 2009).

Similarly, the findings of this study report that the majority of ECP participants were employed in the private sector. Factors such as high patient loads, long working hours, inadequate resources and occupational hazards are some of the reasons cited by healthcare professionals for avoiding employment in the public health system (George & Reardon 2013). It is, therefore, plausible that this might be the case for the ECPs who participated in this study too.

One of the dominant themes identified from the participants’ responses with regard to the types of human errors that occur in the pre-hospital emergency care setting, is ‘poor judgement error’, which was perceived as diagnostic and medication errors. Diagnostic errors were identified as causing one of the major adverse events that patients experience in clinical practice (Neale, Hogan & Sevdalis 2011).

While the actual diagnostic error rates in clinical practice are difficult to determine, it has been estimated that about 10% – 15% of all rendered diagnoses are incorrect (Graber 2013). In the pre-hospital emergency care setting, diagnostic errors are recognised as the most frequent causes of allegations of negligence, and contribute to a worldwide high mortality rate every year (Pelaccia, Messman & Kline 2020). This suggests that diagnostic errors might be responsible for some of the mortality recorded in the pre-hospital setting in South Africa (Meel 2004).

An accurate diagnosis by a health care practitioner is essential for directing immediate treatment and subsequent care, and can improve the patient’s outcome (Koivulahti, Tommila & Haavisto 2020). Administering medications urgently during emergency care makes the process prone to error. Our finding, namely, that administration of incorrect drug dosages is a type of human error that occurs in the pre-hospital setting, is consistent with findings of the literature (Nguyen 2008).

Factors such as poor medication knowledge, wrong calculations, and nomenclature issues have been attributed to medication error (Lesar, Briceland & Stein 1997). It is, therefore, plausible that such factors may underlie the medication error as perceived by the participants of this study.

Bijani et al. (2021) report that good clinical knowledge, experience, and skills contribute to an ECP’s professional capabilities, which are essential for making good clinical decisions and avoiding error (Bijani et al. 2021). This suggests that an opposing state, that is, poor skills and knowledge, and limited experience, can contribute to error during pre-hospital care. To this end, participants listed ‘practitioner’s incompetence’ as factors that can influence patient safety in the pre-hospital setting.

In addition, participants identified ‘Insufficient clinical knowledge and experience’ and ‘Insufficient education and training’ as factors that are perceived to contribute to error during pre-hospital emergency care. These factors were found to correlate positively with ‘Poor skill or knowledge-based error’, and suggests that insufficient clinical knowledge, and too little education and experience precipitate poor skill or knowledge-based errors. This finding highlights the need for adequate and effective education and training.

Fatigue is a complex phenomenon that affects physical characteristics, cognition, behaviour and mental health of an individual (Ramey et al. 2019). A growing body of evidence suggests that fatigue can negatively affect a health care practitioner’s performance. Increased levels of fatigue are associated with poor cognitive function (Harrison & Horne 2000), impaired performance, and increased error and accident rates (Ramey et al. 2019). Similarly, participants of this study reported ‘work related fatigue and/or stress’ as a factor that can contribute to perceived error in the pre-hospital emergency care setting.

Work-related fatigue and/or stress correlates positively with ‘Fatigue-related error’, a type of error identified by the participants. Moreover, participants listed fatigue and stress as factors that can influence patient safety in the pre-hospital setting. Fatigue management approaches, such as limiting shift length and promoting adequate rest between shifts, can be used to combat fatigue-related error.

Poor communication is an important cause of adverse events in healthcare system, and can result in medical error (Bari et al. 2016). Likewise, participants in our study reported that ‘poor communication skill’ can contribute to errors, and this was found to correlate positively with ‘communication error’ in the pre-hospital emergency care setting. It has been suggested that routine team checklist briefing can help to improve team communication (Bari et al. 2016).

Our findings also reveal that participants identified ‘individual error’ as a type of error in the pre-hospital care setting. As the participants indicated, individual error was associated with negative behaviours and emotions. In their study that investigated causes, consequences, and emotional responses to medical errors, Bari et al. (2016) report that lack of knowledge is often associated with negative emotions, such as fear and lack of confidence, and could lead to human error (Bari et al. 2016) – again emphasising the role of adequate education and training in averting human error in the pre-hospital care setting. Overconfidence has been identified as a source of cognitive bias that may influence reasoning and medical decision-making, and can cause errors (Berner & Graber 2008). Similarly, participants of this study reported that being overconfident is a factor that can contribute to errors in the pre-hospital care setting.

While they are on duty, ECPs in South Africa often suffer physical abuse, are assaulted and subject to violent behaviours by patients, bystanders and criminals (Holgate 2015). Emergency medical service personnel have been robbed, assaulted or shot at in the line of duty (Seleka 2021). The constant fear for their own life and personal safety experienced by these ECPs can lead to error and adverse events relating to patients. Hence, participants of this study reported that ‘unsafe work environment’ and ‘personal safety concerns’ could contribute to error and influence patient safety in the pre-hospital emergency care setting.

Including hostile environment awareness training (HEAT) programmes in the undergraduate emergency medical care curriculum has been suggested as a strategy to enable ECPs cope with hostile environments (Vincent-Lambert & Westwood 2019). Hostile environment awareness training programmes focus on developing an individual’s ability to assess situations, recognise specific risk factors, and, where possible, avoiding these risks (Vincent-Lambert & Westwood 2019). The role of health leadership should be centred on identifying priorities and providing strategic direction in order to improve health services (Reich, Javadi & Ghaffar 2016).

To improve patient outcomes in the pre-hospital setting, ECPs must demonstrate competence regarding both effective leadership and clinical leadership skills (Martins et al. 2018). To this end, ECPs who participated in this study recognised ‘poor leadership and management practices’ as factors that could contribute to errors during pre-hospital emergency care. The hierarchical parameters entrenched by the level of qualification and training in the pre-hospital environment ensures that the followership role is often assigned to lower-level cadres, while practitioners with higher qualifications assume leadership roles (Rowland, Adefuye & Vincent-Lambert 2021). This practice can lead to intimidation by and unsolicited pressure from colleagues, which can be a source of errors during pre-hospital emergency care, as indicated by the participants of this study.

The authors’ finding that the participants of this study perceived inadequate equipment and environmental issues as factors that could influence patient safety in the pre-hospital emergency care setting, is consistent with similar findings by Guise et al. (2015). Participants highlighted how a shortage of proper tools or equipment to undertake job functions can influence patient’s safety negatively. Environmental factors such as hazardous scenes, crowds, the weather, and crime are also some of the factors reported by the participants to influence patient safety. This is consistent with similar findings by Vincent-Lambert and Mottershaw (2018).

Teamwork between pre-hospital emergency care providers and other healthcare professionals is essential for managing acute clinical emergencies. It has been reported that inadequate teamwork is often responsible for preventable medical errors (Aron & Headrick 2002; Hughes et al. 2016; Rosen et al. 2018). Similarly, participants of our study reported that ‘ineffective teamwork’ could influence patient safety in the pre-hospital emergency care setting. It is recommended that deliberate teaching and training on principles and practice of crisis resource management is undertaken to enhance teamwork skills of health care practitioners (Rowland et al. 2021).

Medical error is a serious public health problem and a leading cause of death globally. It is not easy to find the consistent cause of errors and, even if the cause is found, providing a consistent and viable solution that minimises the likelihood of recurrent errors is difficult (Rodziewicz, Houseman & Hipskind 2018). Healthcare providers and institutions have been encouraged to implement protocols and processes to reduce medical error (La Pietra et al. 2005). In this study, the majority of the participants who worked in the public sector indicated that they do not have a protocol for managing medical error at their workplace, nor have they ever received training on patient safety. We, therefore, advocate that public emergency care practitioners in South Africa establish a culture of safety that focuses on system improvement to overcome errors in the pre-hospital emergency care setting.

## Conclusion

In conclusion, this study investigated health care practitioners’ views on types of errors that occur in the pre-hospital emergency care setting, as well as factors that influence patient safety and precipitate errors during pre-hospital care. According to the participants of this study, errors in the pre-hospital emergency care setting can be categorised under five themes, namely, poor judgement error, poor skill/knowledge-based error, fatigue-related error, individual error, and communication error. Work related fatigue and/or stress, insufficient education and training, insufficient clinical knowledge and experience, and unsafe work environment are some of the factors that contribute to human error during pre-hospital care. The authors identified inadequate equipment, environmental factors, personal safety concerns and practitioners’ incompetence as the four major influencers of patient safety in the pre-hospital emergency care setting. Furthermore, the study found that public-sector EMS in South Africa seldom train the ECP on patient safety or have a protocol for managing medical error. The authors propose that implementing strategies that enhances education and training, clinical skill development, teamwork skills, fatigue management, and leadership skills can help prevent some of the errors identified. In addition, simple practices, such as implementing viable safety protocols to prevent and manage medical error and ensuring that ECPs are continuously trained on maintaining patient safety during pre-hospital care can also assist in this regard.

## Limitations

A major limitation of this study was that some participants did not answer all the questions in the questionnaire. However, this did not compromise the validity and reliability of the findings of this study as responses obtained for most subset of questions surpassed the estimated minimum sample size calculated for this study.
